# Supplementary figures and images for: Domesticated Populations of Codium tomentosum Display Lipid Extracts with Lower Seasonal Shifts than Conspecifics from the Wild—Relevance for Biotechnological Applications of this Green Seaweed
Source: Mar Drugs. 2020 Mar 31;18(4):188. doi: 10.3390/md18040188 (PMC7230330; doi:10.3390/md18040188)

a.

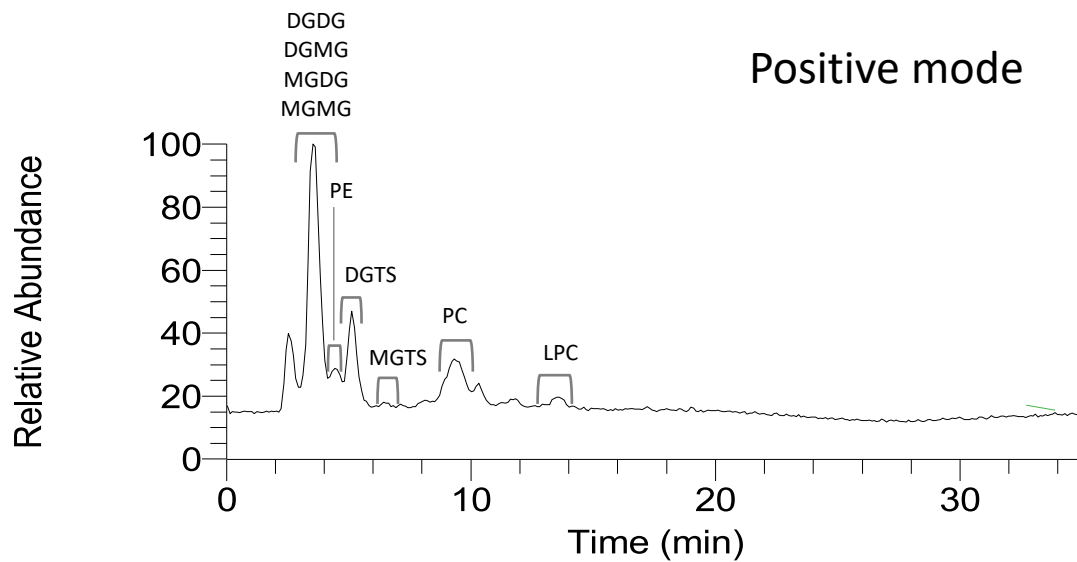

b.

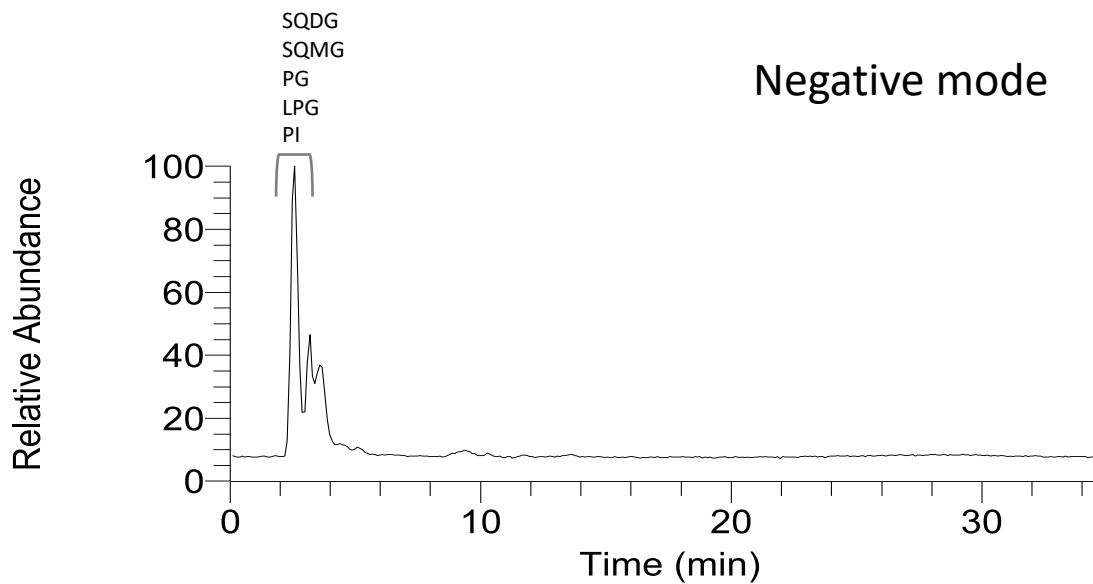

Supplement: Supplementary file 1 [file marinedrugs-18-00188-s001.zip › Supplementary_Files/Supplementary_Figure_S1.pdf]

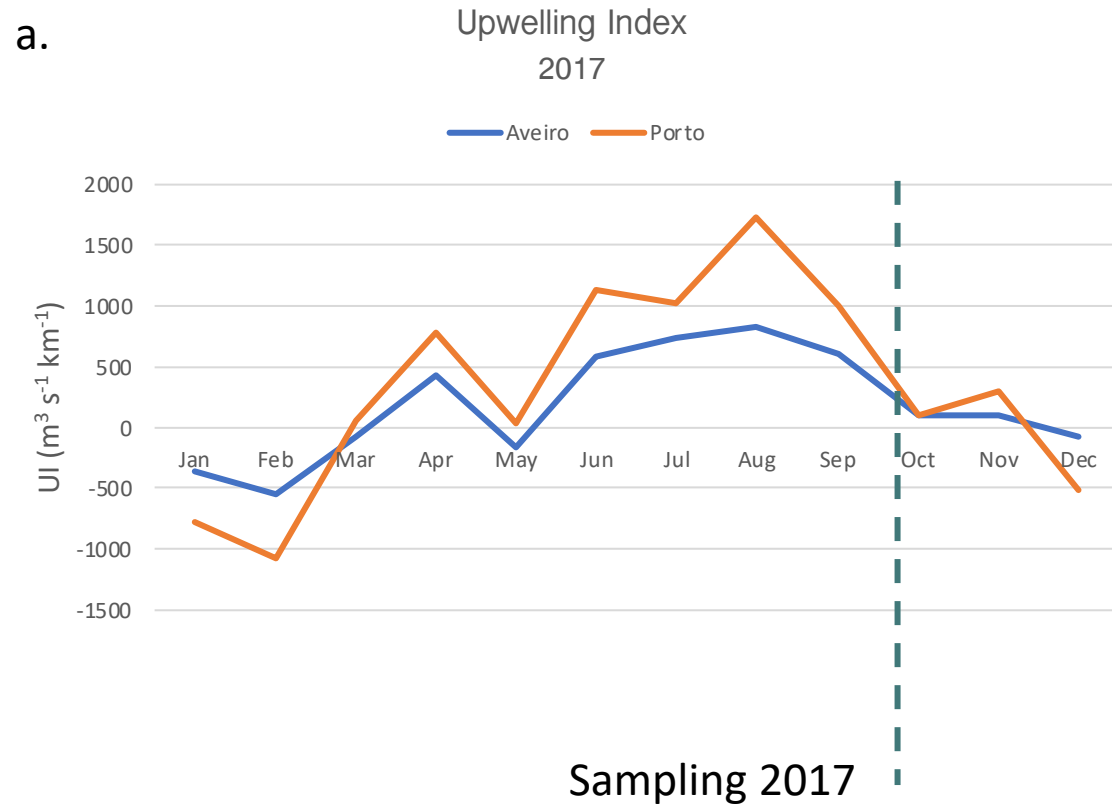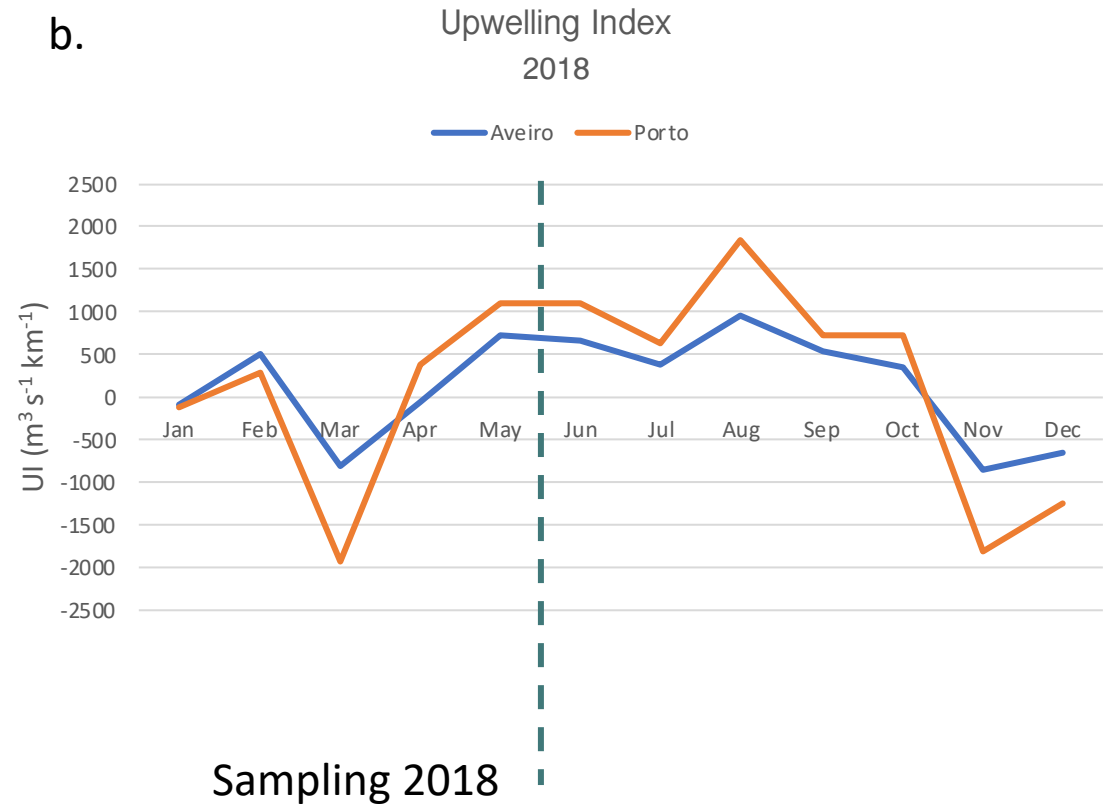

Supplement: Supplementary file 1 [file marinedrugs-18-00188-s001.zip › Supplementary_Files/Suplplementary_Figure_S2.pdf]
